# Effect of drought and nutrient availability on invaded plant communities in a semi‐arid ecosystem

**DOI:** 10.1002/ece3.9296

**Published:** 2022-09-09

**Authors:** Hamada E. Ali, Solveig Franziska Bucher

**Affiliations:** ^1^ Botany and Microbiology Department, Faculty of Science Suez Canal University Ismailia Egypt; ^2^ Department of Biology, College of Science Sultan Qaboos University Muscat Oman; ^3^ Institute of Ecology and Evolution with Herbarium Haussknecht and Botanical Garden, Professorship of Plant Biodiversity Friedrich Schiller University Jena Jena Germany; ^4^ German Centre for Integrative Biodiversity Research (iDiv) Halle‐Jena‐Leipzig Leipzig Germany

**Keywords:** global change, invasion success, invasive plant species, plant functional traits

## Abstract

Ecosystem functions are heavily dependent on the functional composition of the plant community, i.e., the functional traits of plants forming the community. This, on the one hand, depends on plant occurrence, but on the other hand, depends on the intraspecific variability of functional traits of the species, which are influenced by climate and nutrient availability and affected by plant–plant interactions. To illustrate that, we studied the effects of drought and nitrogen addition (+ N), two important abiotic variables which are changing with ongoing global change, as well as their combined effect on the functional responses of grassland communities in semi‐arid environments of Northern Africa comprising of natural and invasive species. We conducted an experiment where we planted three native species and one invasive plant species in artificial communities of five individuals per species per plot. We exposed these communities to four different treatments: a drought treatment, an N‐addition treatment, the combination between drought and N‐addition, as well as a control. To assess the performance of plants within treatments, we measured selected plant functional traits (plant height, specific leaf area [SLA], leaf dry matter content [LDMC], N content of the leaves [N_mass_], specific root length [SRL], and root diameter) for all individuals occurring in our plots, and additionally assessed the above and belowground biomass for each plant individual. We found that the invasive species showed a higher performance (higher biomass accumulation, taller plants, higher SLA, N_mass_, SRL, and root diameter as well as lower LDMC) than the native species under drought conditions. The invasive species was especially successful with the combined impact of drought + N, which is a likely scenario in ongoing global change for our research area. Thus, plant functional traits might be a key factor for the invasion success of plant species which will be even more pronounced under ongoing global change.

## Graphical abstract

Climate, nutrient availability, and invasive plants are major threats to ecosystems. The study measures the invasion success of *Imperata cylindrica* under climate change and nitrogen application. The study found that the invasion success increased under drought conditions. This invasion success was promoted by plant functional traits.

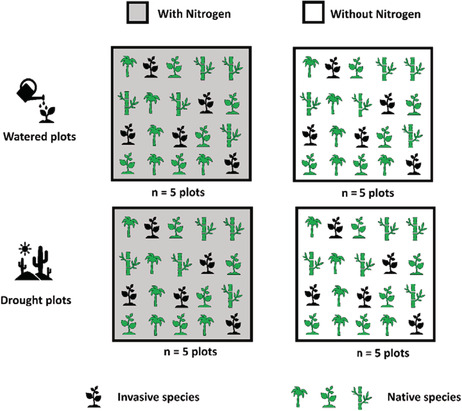

## 1 INTRODUCTION

One of the major drivers of ecosystem functions and compositions is the ongoing global change, which encompasses climate as well as direct anthropogenic impacts (Shi et al., 2018; Zhu et al., 2016). Most research in this area focuses on the effects of temperature, which is generally increasing over the last decades (IPCC, 2021). Especially in arid and semi‐arid ecosystems, an increase in drought events (both in severity and frequency) and the input of N in these nutrient‐poor environments resulting from increased N depositions are of major importance (Dai, 2012; Galloway et al., 2008; IPCC, 2021; Sala et al., 2000; Yang et al., 2018). These changes force plant species to adjust to their new environments (either via plastic responses or via adaptations, i.e., shifts in the genetic structure), otherwise, they are lost from the local species pool (Hoffmann & Sgrò, 2011). The changes in precipitation and the occurrence of drought events have an impact on soil water availability, which may lead to a decrease in ecosystem structure and function (Germino et al., 2015). The increase in nutrient availability due to fertilizer spillover from agriculture into natural areas in addition to increasing fluctuations of extreme weather events (e.g., droughts) will consequently affect nutrient cycling by increasing both, the overall N deposition in the landscape as well as the temporal variability in nutrient availability for plants growing in natural environments (Davis & Pelsor, 2001; Mahood et al., 2022; Verma & Jayakumar, 2012). One of the major aims of global change research is to understand the effects of these changes in abiotic conditions on plant species composition as well as the functional composition of the communities (Cardinale et al., 2012).

Another major influence on the natural ecosystem which is increasing with the ongoing global change is the occurrence of invasive species (Bradley et al., 2012; Seebens et al., 2015). The decrease in ecosystem structure and function through drought events facilitated plant invasion success (Davis et al., 2000; Duell et al., 2021; Leal et al., 2022). For example, during drought events, the biomass production of native species is often reduced, whereas invasive species show high growth rates, especially in nutrient‐poor soils (Pellegrini et al., 2021; Sardans et al., 2017). High niche overlap which describes whether co‐occurring species share parts of their niche space with each other (Harrison et al., 2010) might lead to competition and/or exclusion of some species (Liu et al., 2020). On the other hand, low niche overlap indicates lower levels of interaction, thereby allowing the sustainable co‐existence of species (Kim & Ohr, 2020). The degree of niche overlap of functional traits among plant species strongly influences invasion success and coexistence, as invaders can be more competitive in exploiting shared and limited resources (Kumschick et al., 2014). This resource exploitation is always based on resources available; as in resource‐rich environments, invasive plants tend to have higher growth rates assessed via higher specific leaf area (SLA) and leaf nitrogen content (N_mass_) than native species within the same sites (Allison & Vitousek, 2004; MacDougall et al., 2009; Van Kleunen et al., 2010), while in low‐resource environments, invasive species tend to possess resource acquisition strategies (Funk, 2013; Gioria & Osborne, 2014). Finally, niche overlap with a strong invasive competitor is likely to have strong direct effects on the resident species, e.g., via competitive displacement (Hooper & Dukes, 2010; Ni et al., 2021; Vitti et al., 2020).

Measuring the invasion success of plants can be achieved using several methods based on the scale of the study; for large‐scale studies, invasion success can be assessed by measuring the successful spread of plants from sites of the initial establishment to the surrounding environments (Richardson et al., 2000), and for small‐scale field studies, it is possible to assess the invasion success on the individual level as the increase in the productivity of the invader (e.g., biomass production) in comparison to the native species (Duell et al., 2021; Shi et al., 2021). Understanding the interplay between climate changes in the form of droughts, nutrient input, and biological invasion is critical for predicting the consequences of changes in functional composition on ecosystem functions (Bernhardt‐Römermann et al., 2011; Boscutti et al., 2020; Pejchar & Mooney, 2009).

Ecosystem functions are dependent on the functional composition of the plants therein (Cadotte, 2017; de Bello et al., 2010). The functional composition refers to morphological and eco‐physiological characteristics, so‐called plant functional traits of the plant species or individuals occurring in the ecosystem (Leps et al., 2006; Violle et al., 2007, 2012). Plant functional traits are important as they help track environmental changes, determine ecosystem functioning, and help understand mechanisms that shape species occurrence patterns within ecosystems and their response to global change (Campetella et al., 2020; Liu et al., 2021). Moreover, as plant traits can respond to the environment, they also affect the biotic and abiotic environment by changing soil physical properties and soil biota through their root systems (Maire et al., 2009) or enriching soil N through symbiotic N_2_ fixation (Herridge et al., 2008). There are thus effect and response traits, as traits that are identified as response traits may at the same time affect the environment and resources of other species (Violle & Jiang, 2009). The functional composition of ecosystems, on the one hand, depends on the nature of plant species comprising these ecosystems, but on the other hand, depends on the variability of plant functional traits within species which is caused by variations in abiotic and biotic factors (Albert et al., 2010; Ali & Bucher, 2021; Bucher et al., 2016; Rosbakh et al., 2015; Violle et al., 2012).

We selected eight parameters capturing competition and performance of plants, namely, above and belowground biomass, the maximum height of the plants (H_max_), specific leaf area (SLA), leaf dry matter content (LDMC), leaf nitrogen content (N_mass_), specific root length (SRL), and root diameter for this study (Pérez‐Harguindeguy et al., 2013). Competition is best captured with plant biomass, both above and belowground, as well as H_max_ as competition for light is the most dominant factor (Pérez‐Harguindeguy et al., 2013). SLA is also a measure of competitive strength and is mainly related to growth rates (Garnier et al., 1997; Hulshof et al., 2013; Knops & Reinhart, 2000; Pérez‐Harguindeguy et al., 2013). LDMC is a measure of investment of the plant species in defense and structural components (Pérez‐Harguindeguy et al., 2013). Leaf nitrogen scales with photosynthesis rates as most N in the leaves is located in rubisco, the main enzyme of carbon fixation (Evans, 1989). Specific root length, which is the measure of the length‐to‐mass ratio of the roots, is one of the most commonly measured morphological parameters of roots as it characterizes the economic aspects of root systems (Ostonen et al., 2007). The root length is assumed to be proportional to resource acquisition (benefit), whereas the root mass relates to construction and maintenance (cost) (Fitter, 2002; Fitter et al., 1996; Ostonen et al., 2007). Finally, we studied the root diameter, which not only determines the surface area of direct interaction between roots and soil but also the root surface area colonized by mycorrhizal fungi assisting in plant nutrient acquisition and drought resistance (Comas et al., 2013).

The current study focused on semi‐arid grasslands in Northern Africa and the impact of these major threats, namely, drought, and N‐addition on plant invasion success. We tested the effect of these parameters on the functional response of native and invasive species in a pot experiment, where plants were exposed to drought, N‐addition, or a combination thereof. We used species from semi‐arid grasslands in Egypt as previous studies suggested that Egypt is facing extreme drought events during the coming decades (Asklany et al., 2011; Mossad & Alazba, 2015). To our knowledge, this is the first study that looked at the combined effects of drought and N‐addition on the invasion success of an invasive species in a semi‐arid ecosystem. We hypothesized that:

1. As there is a strong species‐specific response to drought and N‐addition and native species are maladapted to changed environmental conditions, there is an increase in invasion success in terms of biomass of *I. cylindrica* as invasive species outcompete native species (Hypothesis 1).
2. This positive effect of invasive plants is mainly promoted functionally by several plant response traits which are related to competitive ability and performance (e.g., H_max_, SLA, LDMC, N_mass_, SRL, and root diameter) where we expect to see changes in the native and invasive species in the mixed cultivation as compared to the plots, where they are not grown together (Hypothesis 2).

Knowing the response of the functional composition of a community to changes in abiotic factors (e.g., drought and N‐addition) as well as biotic changes through the occurrence of invasive species may allow a general understanding of vegetation responses to global change and associated consequences for ecosystem productivity (Alexander & Levine, 2019; Manzoor et al., 2021).

## 2 METHODS

### 2.1 Study area

Seeds and soil used in the experiment were collected from the Lake Manzala coast (31°15′N, 32°11′E), which is located in the North of Egypt. The area has a Mediterranean climate that is hot and dry in summer and wet and warm in winter, with a minimum temperature of 12.5°C in January and a maximum of 30°C in August. The total rainfall is 100 mm per year, mostly between November and February (Ahmed et al., 2009). The soil has a coarse texture with high amounts of sand and gravel, low pH, and high CaCO_3_ (Elnaggar & El‐Alfy, 2016).

### 2.2 Study species

We focused on the three native species, *Cakile maritima* Scop., *Fumaria densiflora* DC., and *Ranunculus sceleratus* L., and the invasive species *Imperata cylindrica* L., which were planted together in artificial communities. These four species were chosen based on their occurrence in the natural habitat according to a previous study (Ali & Bucher, 2021) to imitate the natural plant communities found in the field. *I. cylindrica* is a perennial rhizomatous grass belonging to the Poaceae family, it is highly flammable, and mainly native to tropical and subtropical Asia and is considered a weedy pest in 73 countries worldwide (MacDonald, 2004). It invades not only crops but also grassland ecosystems in Egypt, causing serious economic and environmental damage (Zahran & Willis, 2008).

### 2.3 Experimental setup

**FIGURE 1:**
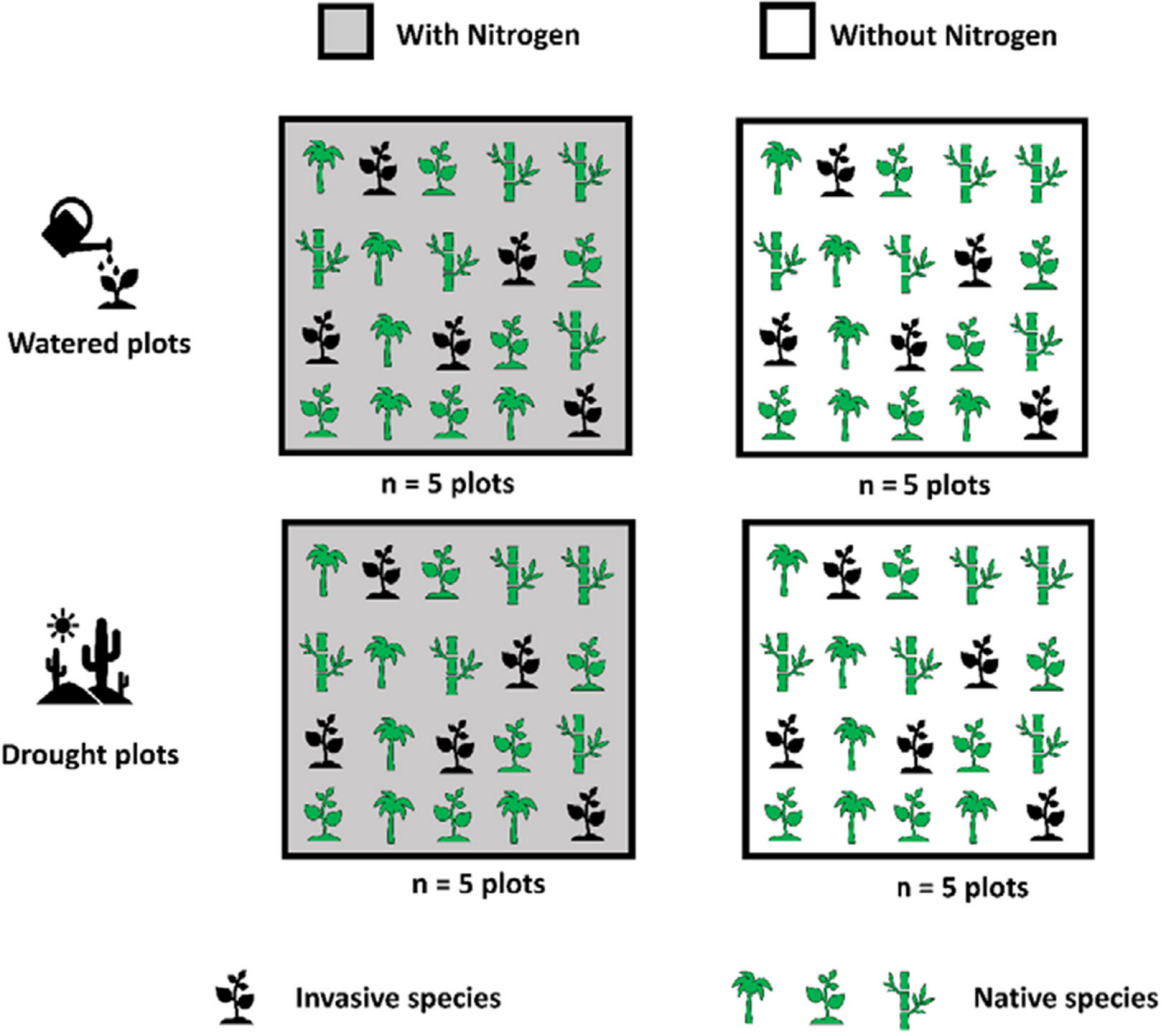
Scheme of the experimental design. A total of 20 plots were cultivated, each plot has four species (one invasive [black] and three native species [green]) with five individuals per species (*n* = 20 individuals/plot). The treatments were control, drought, N‐addition, and drought + N (*n* = 5 plots per treatment).

In the summer of 2019, seeds were collected from the study area and stored at −20°C. We set up four experimental treatments, namely, control, drought, N‐addition, and drought + N. A total of 20 plots were cultivated, i.e., every treatment and the control were repeated five times (Figure 1).

The seeds were sown in soil collected from the study area during the seed collection on March 1, 2020. Before the experiment, the soil was sieved with a 10 mm sieve, mixed throughout, and sterilized twice for 150 min at 80°C (Dietrich et al., 2020), and filled into 50 × 50 cm permanent plots in the ground to the depth of 25 cm; finally, a layer of fresh soil that was not sterilized was added at the top of each plot (5 cm), the plots were 1 m apart from each other. We calculated the amount of water needed to saturate the experimental plots by watering five of the plots for 5 days. Nine hundred milliliter of water was needed to saturate the experimental plots. All the plots were watered daily with 540 ml (60% of water saturation) during the first week, then they were watered every second day from week 2 until week 4. On April 16, 2020, five similarly sized individuals per species were chosen for the experiment (*n* = 20 individuals per plot, 15 native individuals [five each per species], and five individuals of *I. cylindrica*); the additional seedlings, as well as any other species grown within the study plots, were removed at the beginning of the experiment.

In order to simulate the effect of drought, the experimental plots were divided randomly into two treatments. While half of the plots (*n* = 10) were watered with 540 ml twice a week, the drought plots (*n* = 10) were also watered twice a week but with just 180 ml, which represents 20% of soil saturation. To a random subset of five of the dry plots and five of the control plots (*n* = 10), 100 mg of ammonium nitrate (NH_4_NO_3_) containing 33.5 mg of N was applied at weekly intervals for a total of 10 weeks (from April 16th to July 5th) to simulate the nitrogen input applied to this type of soil as recommended for agricultural use by Elrys et al. (2019). Our experiment lasted for 158 days from the first day we added the treatments until the harvest. Planting, watering, and harvesting of the plots happened at the same time for all treatments and the control.

### 2.4 Data collection

All traits were measured using the standard methods described by Pérez‐Harguindeguy et al. (2013) on each individual within each plot (*n* = 400) to account for intraspecific trait variability (Albert et al., 2012; Ali et al., 2017). Before harvest on September 20, 2020, the selected above and belowground traits (above and belowground biomass, H_max_, SLA, LDMC, N_mass_, SRL, and root diameter) were measured (Pérez‐Harguindeguy et al., 2013). H_max_ (cm) was measured as the shortest distance from ground level to the highest photosynthetic tissue using a ruler. To measure SLA and LDMC, three healthy fully developed and sun‐exposed leaves were collected for each individual in each plot and measured together as one pooled sample. SLA, which is defined as the ratio of fresh leaf area (LA) to dry mass expressed as (mm^2^/mg), was measured by measuring the two leaf dimensions using a ruler (mm), then these two dimensions were multiplied to get the total LA (mm^2^). The leaves were weighed to record the fresh mass and subsequently oven‐dried at 70°C for 48 h and weighed again to assess the leaf dry mass (mg). Finally, the LA was divided by the leaf dry matter to calculate SLA. In addition to that, LDMC was measured as the dry mass (mg) divided by its water‐saturated fresh mass (g), expressed in mg/g. Moreover, we measured the leaf nitrogen percentage on the same oven‐dried leaves that were used for measuring the SLA and LDMC, using the Semimicro–Kjeldahl method (Bremner, 1996). For biomass harvest, invasive and native species were cut at the soil surface, dried at 70°C for 48 h, and weighed as aboveground biomass (g). The invasion success of *I. cylindrica* was assessed as biomass fraction per plot by dividing the biomass of *I. cylindrica* by the total biomass of all plants in the community (Shi et al., 2021); this method reflects whether *I. cylindrica* outcompeted the native species in terms of productivity. Moreover, the whole root system was carefully pulled out of the soil, washed, dried, weighted, and scanned with a flat‐bed scanner at a resolution of 800 dpi. RhizoVision Explorer was used to measure the root length (cm) and root diameter (cm) for each individual using the scanned images (Seethepalli et al., 2021). Additionally, we measured SRL (cm/g); expressed as root length divided by the root dry mass.

### 2.5 Data analysis

To analyze the differences in the above and belowground biomass, the plant functional traits of the native and invasive species, and the invasion success of *I. cylindrica* in each treatment, we used pairwise comparisons using Tukey's test (Crawley, 2012). Moreover, linear mixed‐effects models (LMM) were used to analyze two main responses: (1) the effect of drought and nutrient enrichment on the invasion success of *I. cylindrica* (above and belowground biomass) (Hypothesis 1). Here, we used the shoot and root biomasses as response variables, and (2) the functional response of plant traits to drought and nutrient enrichment to test which traits are responsible for the invasion success (Hypothesis 2). In these models, biomass (above and belowground), H_max_, SLA, LDMC, N_mass_, SRL, and root diameter were used as response variables. In all cases, the interaction between treatments (control, drought, N‐addition, and drought + N) and species origin (native vs. invasive) were used as fixed variables, and the plot ID was used as a random intercept. The parameter estimates were calculated with restricted maximum likelihood (REML). Finally, we compared the marginal and conditional *R* ^2^ for each model to assess the impact of the plots (Nakagawa & Schielzeth, 2013).

All statistical analyses were performed with R, version 4.0.2 (R Development Core Team, 2022), the LMMs were performed using the package “*nlme*” (Pinheiro et al., 2022) and Tukey's pairwise comparison was done using package “*emmeans*” (Lenth, 2022).

## 3 RESULTS

### 3.1 Effects of drought and N‐addition on the performance of invasive vs. native species

**FIGURE 2:**
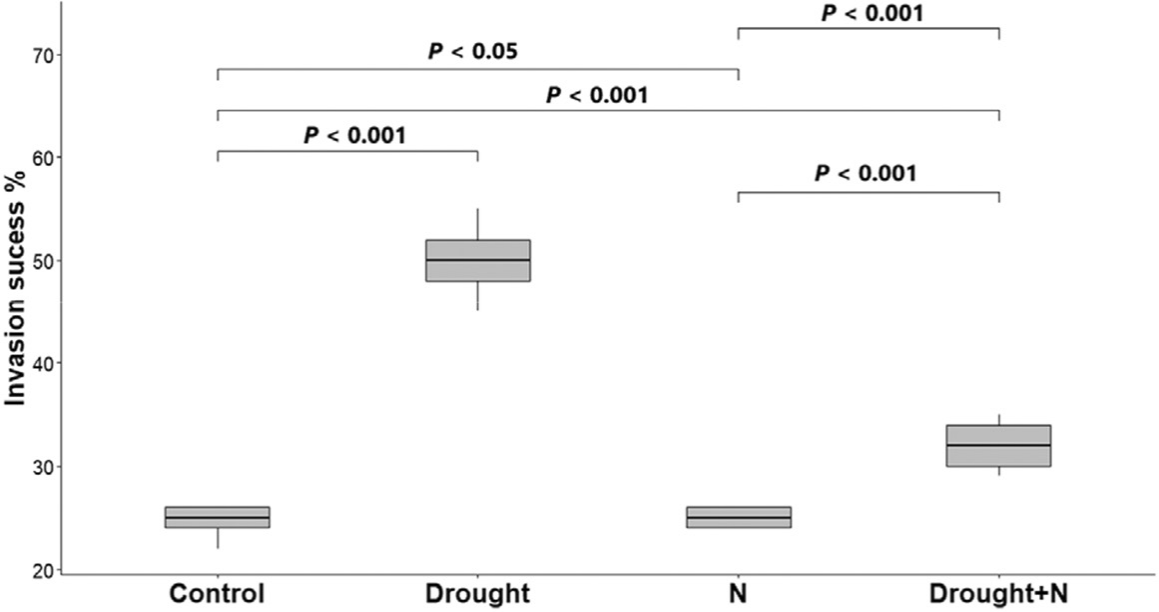
Invasion success measured via the percentage of *Imperata cylindrica* under drought and nutrient application as compared with the control treatment. Numbers are *p* values of the statistically significant differences between indicated groups based on pairwise comparisons using Tukey's multiple‐comparison test.

Overall, the invasion success of *I. cylindrica* assessed via the difference in biomass production between native and invasive species was significantly higher in the drought treatments (+25.36%, *p* < .001, Figure 2) followed by drought + N‐addition (+17.94%, *p* < .001, Figure 2) in comparison to the control plots, but this increase was partially significant between control and N‐addition plots (+0.4%, *p* < .05, Figure 2).

**FIGURE 3:**
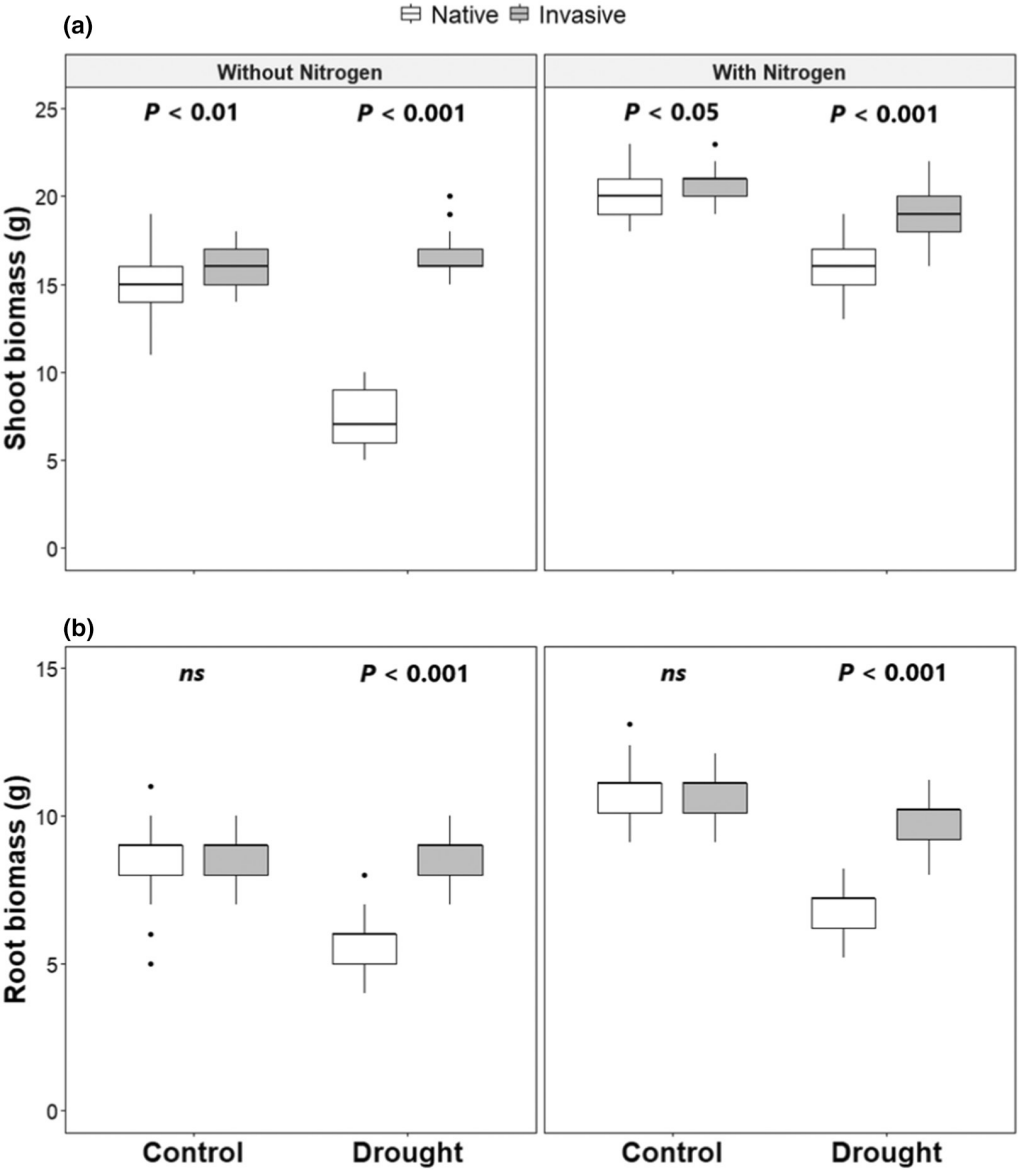
Effect of drought and nutrient application on (a) shoot biomass and (b) root biomass of invasive and native species. Numbers are *p* values of the statistically significant differences between indicated groups based on pairwise comparisons using Tukey's multiple‐comparison test (ns, non‐significant differences).

The study showed that the invasive species (*I. cylindrica*) had a significantly higher shoot and root biomass under drought conditions than the native ones (*C. maritima*, *F. densiflora*, and *R. sceleratus*; Figures 3a,b and S1, Table 1), which was also confirmed by LMMs, as the models explained 91% and 77% of the variance for the shoot and root biomass, respectively (Table 1). Moreover, the difference between the marginal and conditional *R* ^2^ was very low confirming that there was a small plot effect. Finally, there was no significant difference between the native and invasive plants found in the control and N‐addition‐only plots (Figures 3a,b and S1, Table 1).

**TABLE 1:** The effect, *p*‐values, marginal, and conditional *R* ^2^ for linear mixed‐effect models testing the effect of drought, nitrogen addition, combined effect of drought and nitrogen addition, and the presence of an invasive plant on the shoot, root biomass, and plant functional traits (H_max_, SLA, LDMC, N_mass_, SRL, and root diameter).

Variable	Effect	*p*	Marginal *R* ^2^	Conditional *R* ^2^
Root biomass				
(Intercept)	+	**<.001**	.78	.78
Drought	−	**<.001**		
Drought + N	−	**<.001**		
N	+	**<.001**		
Invasive	+	.3535		
Drought × Invasive	+	**<.001**		
Drought + N × Invasive	+	**<.001**		
N × Invasive	+	.9738		
Shoot biomass				
(Intercept)	+	**<.001**	.91	.92
Drought	−	**<.001**		
Drought + N	+	**.034**		
N	+	**<.001**		
Invasive	+	**.007**		
Drought × Invasive	+	**<.001**		
Drought + N × Invasive	+	**<.001**		
N × Invasive	−	.498		
H_max_				
(Intercept)	+	**<.001**	.76	.76
Drought	−	**<.001**		
Drought + N	−	.295		
N	+	**<.001**		
Invasive	+	**.019**		
Drought × Invasive	+	**<.001**		
Drought + N × Invasive	+	**<.001**		
N × Invasive	−	.411		
SLA				
(Intercept)	+	**<.001**	.85	.86
Drought	+	**<.001**		
Drought + N	+	**<.001**		
N	+	**<.001**		
Invasive	+	.150		
Drought × Invasive	+	**<.001**		
Drought + N × Invasive	+	**<.001**		
N × Invasive	+	.233		
LDMC				
(Intercept)	+	**<.001**	.80	.79
Drought	−	**<.001**		
Drought + N	−	**<.001**		
N	−	**<.001**		
Invasive	+	.280		
Drought × Invasive	−	**<.001**		
Drought + N × Invasive	−	**<.001**		
N × Invasive	−	**.003**		
N				
(Intercept)	+	**<.001**	.85	.85
Drought	+	**<.001**		
Drought + N	+	**<.001**		
N	+	**<.001**		
Invasive	−	.735		
Drought × Invasive	+	**<.001**		
Drought + N × Invasive	+	**<.001**		
N × Invasive	−	.973		
SRL				
(Intercept)	+	**<.001**	.95	.95
Drought	−	**<.001**		
Drought + N	−	**<.001**		
N	−	**<.001**		
Invasive	+	**<.001**		
Drought × Invasive	+	**<.001**		
Drought + N × Invasive	+	**<.001**		
N × Invasive	−	**<.001**		
Root diameter				
(Intercept)	+	**<.001**	.93	.93
Drought	+	.256		
Drought + N	+	**<.001**		
N	+	**<.001**		
Invasive	+	.182		
Drought × Invasive	+	**<.001**		
Drought + N × Invasive	+	**<.001**		
N × Invasive	−	.737		

*Note*: Statistically significant variables are indicated in bold.

### 3.2 Plant functional trait responses to drought and nutrient addition

**FIGURE 4:**
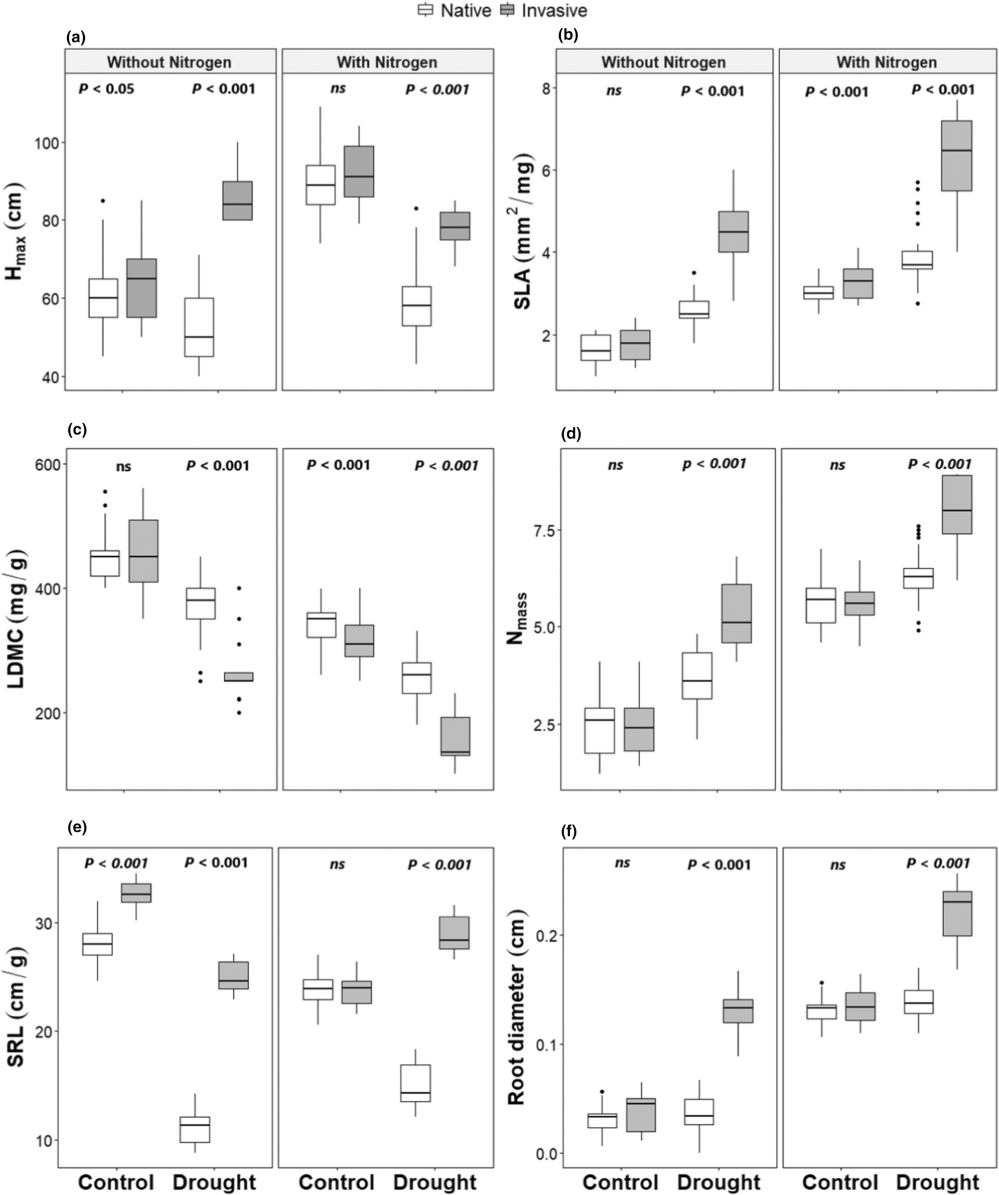
Effect of drought and nutrient application on (a) maximum height of the plants (H_max_), (b) specific leaf area (SLA), (c) leaf dry matter content (LDMC), (d) leaf nitrogen content (N_mass_), (e) specific root length (SRL), and (f) root diameter of invasive and native species. Numbers are *p* values of the statistically significant differences between indicated groups based on pairwise comparisons using Tukey's multiple‐comparison test (ns, non‐significant differences).

Across all the plant functional traits, plots that experienced drought and drought + N showed significant differences between invasive and native species (Figures 4a–f and S2, Table 1). This significant difference was positive in all the studied traits, i.e., invasive species having higher trait values, except in LDMC (Figures 4a–f and S2). For N‐addition plots, the differences between the native and invasive species were significant for only SLA and LDMC, and there was no significant difference between the invasive and native species for the other traits (Figures 4a–f and S2, Table 1). In the control plots, this difference was significant only in SRL (Figures 4e and S2, Table 1) and partially significant in H_max_ (Figures 4a and S2, Table 1). These significant effects were also found in the LMMs, as the models explained 75%, 85%, 80%, 84%, 95%, and 93% of the variance for H_max_, SLA, LDMC, N_mass_, SRL, and root diameter, respectively (Table 1). The difference between the marginal and conditional *R* ^2^ was very low confirming that there was a small plot effect.

Concerning the effect of drought and nutrient addition on the plant functional traits, we found that drought affected shoot biomass, root biomass, H_max_, LDMC, and SRL negatively, SLA and N_mass_ positively, and it had no significant effect on root diameter. The drought + N‐addition affected shoot biomass, SLA, N_mass_, and root diameter positively, whereas root biomass, LDMC, and SRL were affected negatively, and had no significant effect on H_max_. Finally, N‐addition only showed higher shoot biomass, root biomass, H_max_, SLA, N_mass_, and root diameter and smaller LDMC and SRL than the control (Table 1).

## 4 DISCUSSION

Overall, drought promoted the invasion success of *I. cylindrica* in controlled grassland communities in semi‐arid zones, which was manifested by a higher above and belowground biomass of the invasive species under drought conditions and nutrient enrichment. The invasion success of *I. cylindrica* was promoted by several plant functional traits that are linked to productivity (H_max_, SLA, LDMC, N_mass_, SRL, and root diameter).

While the findings showed that the invasive species *I. cylindrica* possessed significantly higher above and belowground biomass in comparison to the native species in dry and fertilized plots, there was no significant difference in the control and N‐addition‐only plots. These findings are consistent with previous research which suggested that the higher biomass production of invasive species under dry conditions is based on several functional traits, such as robust plasticity which changes above and belowground biomass (Funk et al., 2016). Our findings demonstrated that *I. cylindrica* is an aggressive invasive species and comparably drought tolerant as has been found in Kirwan et al. (2021). Due to the higher drought tolerance, plasticity, and resource utilization efficiency, the invasive species *I. cylindrica* benefit more from drought through more efficient use of soil water in comparison to native species (Dong et al., 2014).

In terms of the response of above and belowground biomass under nutrient addition, our current study showed that both invasive and native species respond similarly. This finding is consistent with a study conducted by Dawson et al. (2012). They compared responses to nutrient addition among four types of species in Switzerland and found that invasive plant species did not differ in their performance in response to nutrient addition from the response of native plant species. The same was also found by Liu and van Kleunen (2017) in a study conducted in Germany on 29 terrestrial herbs, as both invasive and native species produce the same amount of biomass under high nutrient availability.

The current study highlighted that while the invasion success of *I. cylindrica* was significantly higher under drought and drought + N‐addition treatments in comparison to the control plots, the invasion success was marginally significant between the N‐addition and control plots. These results indicate that the invasive species *I. cylindrica* recovers from stressful conditions associated with drought more efficiently than the native one, which can be part of its resilience to drought (Leal et al., 2022), suggests that *I. cylindrica* has rapid phenotypic plasticity to adapt to new and heterogeneous habitats (Ni et al., 2021), in addition to its wide environmental tolerance (MacDonald, 2004) and high growth rate under different growing conditions (Ni et al., 2021). These findings suggest that if drought becomes more frequent, *I. cylindrica* is more likely to invade and impact grassland ecosystems.

The success of invasive over native species was manifested in several plant functional traits which allow the invasive species to grow under several harsh conditions like drought and low nutrient availability. The current study showed a significant difference between the invasive and native species for all six traits studied under drought and drought + N‐addition. These findings confirmed that invasive species have higher values in plant functional traits which are associated with high performance (Van Kleunen et al., 2010; Vitti et al., 2020).

H_max_ showed a significantly higher value for invasive species over native ones under both drought and drought + N‐addition. H_max_ was still higher but only marginally significant in the control plots, and not significantly different in the + N plots. This finding is consistent with previous studies which proved that H_max_ is the most important predictor of invasiveness in habitats under stress (e.g., drought; Grotkopp et al., 2002), confirming that stem elongation is assumed to enhance fitness by improving plants' competition for resources, mainly light (Closset‐Kopp et al., 2011).

The same trend was found for SLA, which is related to water‐use efficiency, i.e., individuals with lower SLA typically display higher drought tolerance (Wright et al., 2004). This was found in the current study as the native species showed a lower SLA in the drought plots than the invasive species as a direct response to drought (Wellstein et al., 2017). SLA is also related to photosynthetic capacity, as high SLA species capture more light and enhances the carbon gain per unit leaf area (Gommers et al., 2013), which gives the invasive species further advantage to perform better than the native ones.

The current study found high SLA and low LDMC in drought and drought + N‐addition plots for the invasive species *I. cylindrica*, thus identifying its potential for fast growth and high biomass accumulation (Hodgson et al., 2011). However, low SLA and high LDMC are related to efficient conservation of nutrients, which was found in the control and N‐addition plots, and in the native species in the drought and drought + N‐addition plots, suggesting that the native species can conserve nutrients but cannot utilize it in their growth, such as shown by Zheng et al. (2017).

Studies showed that high N content in the leaves is linked to protein content, especially rubisco in leaves which allows plants to capture more CO_2_ inside the leaf (Evans, 1989; Wright et al., 2004). This finding is consistent with the results, as we found a higher N concentration in the leaves of the invasive *I. cylindrica* species in comparison with the native species under both drought and drought + N‐addition conditions, hinting at higher photosynthesis rates and thus a higher competitive performance.

The study results showed also higher SRL and root diameter for the invasive species in comparison to the native species under drought and drought + N‐addition treatments, confirming the important role of plant roots in plant acclimation and/or adaptation to drought (Zhou et al., 2018). Moreover, it showed that root traits of invasive species enhance the amount of water and nutrient absorption allowing them to improve plant yield under water stress conditions (Comas et al., 2013; Narayanan et al., 2014). This can be discussed based on SRL that summarizes the overall effect of both root diameter and tissue density in terms of root length per dry biomass invested in the tissue (Fitter, 2002) and root diameter which controls the length and surface area of root systems for given biomass allocated to the root system (Fitter, 2002). Consequently, as the invasive and native species showed higher root diameter in the drought + N‐addition treatment in comparison to the other treatments, i.e., thicker and shorter roots, plants probably had fewer fine roots as a response to nutrient enrichment (Grossman & Rice, 2012; Siebenkäs et al., 2015). In the control plots, there was no significant difference between invasive and native species, both of them had high SRL and low root diameter, indicating that plants can efficiently increase hydraulic conductance by increasing surface area, and thus increasing the volume of soil that can be explored for water (Comas et al., 2012; Den Herder et al., 2010).

In sum, the current study revealed that the invasion success of *I. cylindrica* under drought and drought + N‐addition was enhanced by several response plant functional traits that are linked to productivity (H_max_, SLA, LDMC, N_mass_, SRL, and root diameter), while in the control and N‐addition treatments this increase was less pronounced, which might be due to the fact that invasive species perform better than native species due to more efficient resources use (Allison & Vitousek, 2004; Sardans et al., 2017).

## 5 CONCLUSIONS

In the present study, we showed the effects of drought and nutrient application on the performance of invasive and native species using a field experiment in a semi‐arid ecosystem. Drought promoted the invasion success of *I. cylindrica* in grassland communities, which was manifested in higher above and belowground biomass of the invasive species under drought and nutrient addition. This invasion success of *I. cylindrica* was promoted by several response plant functional traits that are linked to productivity (H_max_, SLA, LDMC, N_mass_, SRL, and root diameter).

## AUTHOR CONTRIBUTIONS

**Hamada E. Ali:** Conceptualization (lead); data curation (lead); formal analysis (lead); investigation (lead); methodology (lead); validation (lead); visualization (lead); writing – original draft (lead); writing – review and editing (lead). **Solveig Franziska Bucher:** Formal analysis (supporting); funding acquisition (supporting); visualization (supporting); writing – original draft (supporting); writing – review and editing (supporting).

## CONFLICT OF INTEREST

The authors declare no conflicts of interest in this study.

## Supporting information


Figures S1‐S2

## Data Availability

All data used in this paper are available in Dryad, https://doi.org/10.5061/dryad.b8gtht7g9.
