# Supplementary material for: Effect of drought and nutrient availability on invaded plant communities in a semi‐arid ecosystem
Source: Ecol Evol. 2022 Sep 9;12(9):e9296. doi: 10.1002/ece3.9296 (PMC9463043; doi:10.1002/ece3.9296)
Supplement: Supplementary file 1 — Figures S1‐S2 [file ECE3-12-e9296-s001.docx]

**Supporting information:**


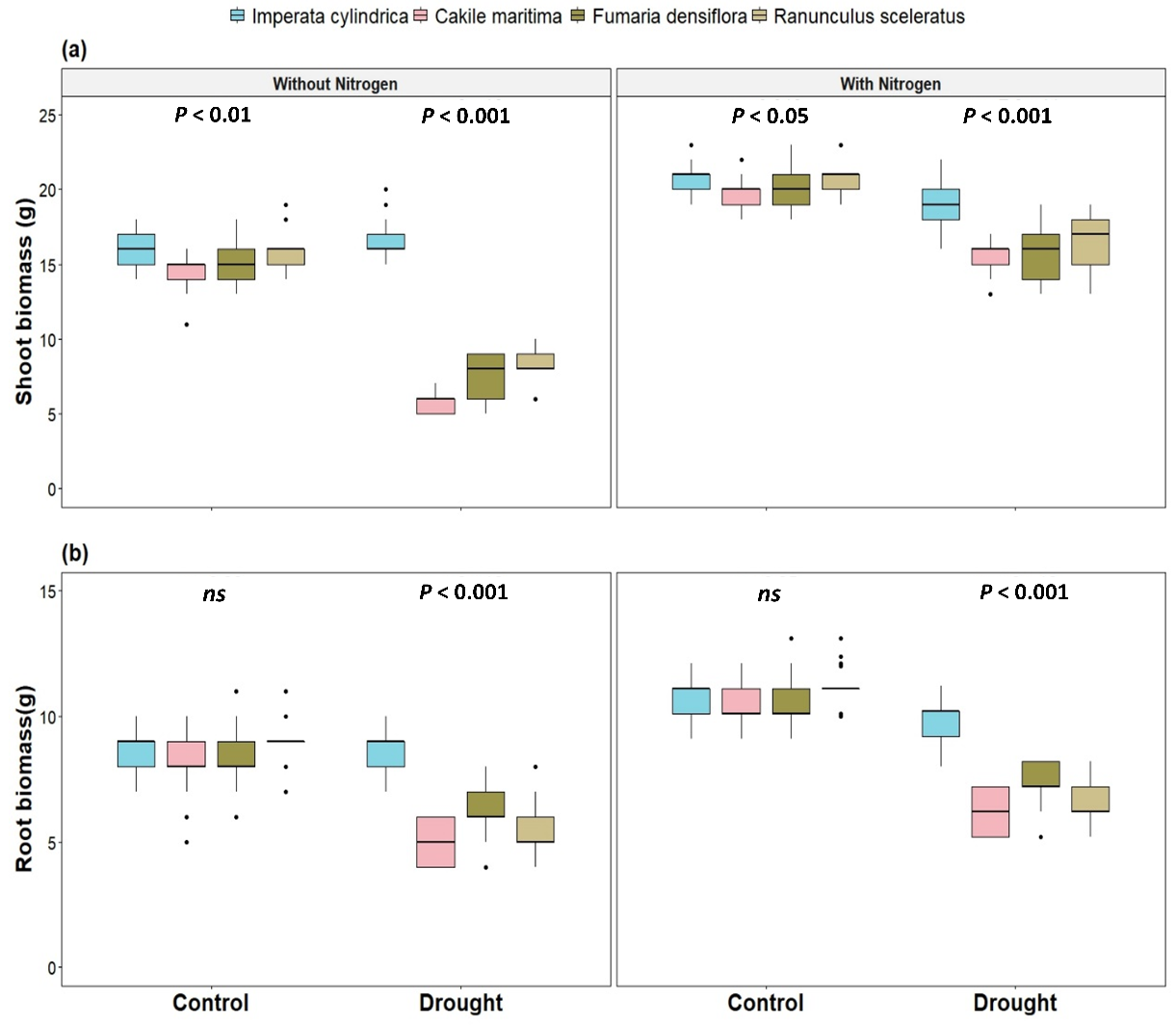


Fig. S1. Effect of drought and nutrient application on (a) shoot biomass and (b) root biomass of invasive and native species. Numbers are *P* values of the statistical signiﬁcant differences between indicated groups based on pairwise comparisons using Tukey's multiple comparison test (ns: non-significant differences).


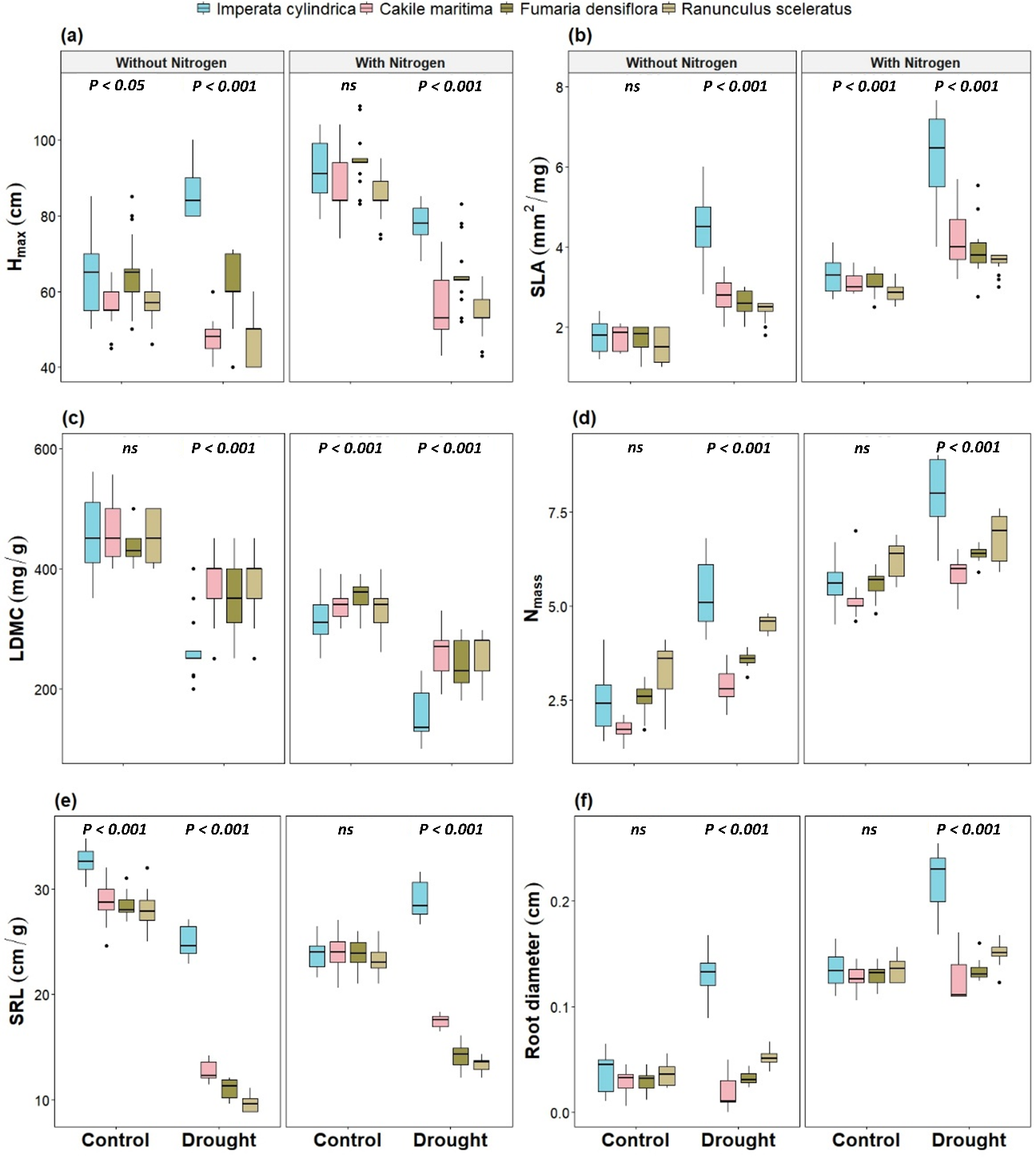


Fig. S2. Effect of drought and nutrient application on (a) maximum height of the plants (H_max_), (b) specific leaf area (SLA), (c) Leaf dry matter content (LDMC), (d) leaf nitrogen content (N_mass_), (e) specific root length (SRL) and (f) Root diameter of invasive (*Imperata cylindrica*) and native species (*Cakile maritima*, *Fumaria densiflora* and *Rananculus sceleratus*). Numbers are *P* values of the statistical signiﬁcant differences between indicated groups based on pairwise comparisons using Tukey's multiple comparison test (ns: non-significant differences).
